# Frequency of nutritional anemia among female medical students of Faisalabad

**DOI:** 10.12669/pjms.332.11854

**Published:** 2017

**Authors:** Shireen Jawed, Sundus Tariq, Saba Tariq, Anwar Kamal

**Affiliations:** 1 Dr. Shireen Jawed, MBBS, M.Phil. Assistant Professor of Physiology, Aziz Fatima Medical College, Faisalabad, Pakistan; 2 Dr. Sundus Tariq, MBBS, M.Phil. Assistant Professor of Physiology, University Medical & Dental College, Faisalabad, Pakistan; 3 Dr. Saba Tariq, MBBS, M.Phil. Assistant Professor of Pharmacology, University Medical & Dental College, Faisalabad, Pakistan; 4 Dr. Anwar Kamal, MBBS, M.Phil. Professor of Physiology, University Medical & Dental College, Faisalabad, Pakistan

**Keywords:** Anemia, Hostelites, Medical students

## Abstract

**Background and Objective:**

Anemia is a common health problem worldwide. This problem is most commonly faced by 18 to 25 years of females. Medical students especially female hostelites poses high risk of anemia because of their poor eating habits, breakfast skipping, long schedule in college, burden of medical studies, clinical postings, and extra-curricular activities. Therefore the current study was designed to determine the hemoglobin status in young female medical students. We also elucidate its association with BMI.

**Methods:**

A cross sectional study was conducted at The University of Faisalabad during December 2015 to February 2016. A total of 221 female students were recruited by convenient sampling technique. All relevant information about participants was taking by administering structured questionnaire. Participants were categorized as hostelities and day scholars for comparison. Study subjects were also sub grouped on the bases of their BMI. Hemoglobin, MCV, MCH and MCHC were estimated at Madina Teaching Hospital Faisalabad. Statistical analysis was performed on SPSS 20.

**Results:**

Mean age of the study subjects was 19.92 ±0.93. 33.4% of the students were found to be anemic. Significantly high number of hostelites (39.2%) were anemic as compared to day scholars (23.1%) (P value= 0.015*). On analyzing by BMI categories, greater number of underweight subjects was found to be anemic as compared to normal and overweight subjects.

**Conclusion:**

Anemia is more prevalent in hostelites as compared to day scholar female medical students which might also affect the efficiency of these students.

## INTRODUCTION

Nutritional anemia is a global problem increasing in developing countries.^1^ This problem is most commonly faced by young females of 18 to 25 years of age. Global Prevalence of iron deficiency anemia was 50% among the females of reproductive age.^2^ Previous documentations in 2015 from Lahore, a city of Pakistan has reported that 50% of the Pakistani females of reproductive age were suffering from anemia and 21% of the females of age group 9-29 years face the same problem in Punjab, a province of Pakistan.^3^ Same prevalence has been reported from neighboring country India.^4^

The main etiological factors responsible for anemia in this age group were age, sex, social class, dietary deficiencies, stress, menstrual blood loss and helminthic infection.^2^,^5^ It was more prevalent among the underweight females having BMI <18.5 kg/m^2^. It may suggest that the nutritional status has a significant reflection upon the prevalence of anemia among the young females. There is evidence that the medical students especially those staying in hostels are at high risk of anemia because of long college schedule, clinical postings, stress and inappropriate diet in their hostels.^6^

Many researchers have reported that anemia is associated with fatigue, general malaise reduced work capacity and poor concentration.^1^ It adversely affects learning, cognitive function, behavior, attention and regular activities of young students^7^,^8^ and may also results in college absenteeism. According to World Health Organization (WHO), anemia is a major public health problem and indicator of poor nutrition and health, which is at its peak in South-East Asia, Eastern Mediterranean and African Regions.^2^,^9^ Study in past revealed that prevalence of anemia was tremendously greater in young girls so additional researches, factors responsible for anemia in young females and public health interventions are required to prevent it. Therefore the current study was designed to see the prevalence of anemia in young female medical students.

Aim of current study was to determine the hemoglobin status in young female university students of age 18 to 25 years and to estimate the severity of anemia among them, as this age group is vulnerable to dietary deficiencies because of their increased physiological needs of micronutrients including iron. We also elucidate its association with BMI.

## METHODS

This cross-sectional comparative study was performed at the Department of Physiology, University Medical and Dental College, The University of Faisalabad during December 2015 to February 2016. A total of 221 female students were recruited by convenient non-probability sampling technique after taking ethical approval from the institutional research and ethic committee. First 300 Students were selected for study.^10^ Information about the participant’s age, education, religion, socio economic status, income of parents, family structure, dietary habits, exercise were included. Questions related to menstruation, like age at menarche, length of cycle, duration of bleeding period, blood loss/ cycle and dysmenorrhea, history of hemoglobinopathies, worm infestation and cardiac diseases were recorded by filling predesigned Performa. Participants with cardiac and pulmonary diseases, renal disorders, hemoglobinopathies, menstrual disorders and history of worm infestation, dehydration due to any cause and on fluid therapy were excluded from the study. Finally, the 221 students were enrolled based on inclusion and exclusion criteria as mentioned.

These students belong to different, cultural and socio economic backgrounds. Written informed consent including objectives and important details of study was taken from each student. Their physical examination was performed to look for signs of anemia and dehydration. Height and weight were measured by stadiometer and BMI was calculated by Quetelet’s index: BMI= weight in kg/ height in m^2^.^11^ Blood sample of each participant was obtained by venipuncture and kept in vacutainers containing anticoagulants for hematological analysis. Hemoglobin levels and red cell indices including MCV, MCH, and MCHC were estimated in Madina Teaching Hospital laboratory by KX-21NTM automated hematology analyzer (Sysmex) which is reliable and give accurate results through the use of automatic floating discriminators. These automated electronic blood cells counter system measures hemoglobin using non-cyanide method which is nontoxic and prevent environmental pollution by cyanide reagents.^12^

The severity of anemia was categorized as per WHO criteria. Hemoglobin levels below 12.0 gm/dl was considered anemia. Hemoglobin levels of 10.0-11.9 gm/dl is graded as mild anemia, hemoglobin 7-9.9 gm/dl and,< 7gm/dl are graded as moderate and severe anemia respectively.^1^ The participants were divide into two groups, hostelites and dayscholars to evaluate which group have higher risk for anemia.

Students were also grouped on the basis of BMI cut off values recommended by WHO for Asians, to check the association of hemoglobin with BMI. Students were classified as underweight (BMI < 18.5 kg/m^2^), Normal (BMI= 18.5-24.9 kg/m^2^) and Overweight (BMI ≥ 25 kg/m^2^).^11^

### Statistical Analysis

Statistical analysis was performed by using the program statistical package for social sciences (SPSS) for window version 20. Descriptive were described in terms of Mean± SD for continuous variables, and frequencies and percentages for qualitative variables. Normality of data was checked by Shapiro-Wilk’s test and Levene’s test. After the assumptions were satisfied parametric tests i.e. Independent sample t test and one-way analysis of variance (ANOVA) were used for comparison of means of study variables (Hb, weight, height, BMI). Post hoc Tuckey’s was applied for multiple comparisons. Proportions were compared using chi square test. P-value ≤ 0.05 was taken as statistically significant.

## RESULTS

Current study comprises of 221 subjects of age 18-25 years with a mean age of 19.9 ± 0.93 years. The descriptive and hematological variables of study population are shown in Table-I. Independent sample t test was used to compare study variables between hostel residents and day scholars of university. Statistically significant differences were noted between the two study groups with respect to age (P value =0.006*), weight (P value = 0.000*), hemoglobin (P value = 0.029*) and MCHC (P value = 0.027*). There were no significant differences in, height, BMI, MCV and MCH of the study population. (Table-II)

**Table-I:** Descriptive Statistics of study population.

*Variables*	*Mean (n=221)*	*Std. Deviation*
Age	19.9231	0.933
Height in inches	62.2751	2.564
Weight in kg	56.5928	9.153
Body mass index (BMI)	22.68	3.671
Hemoglobin in g/dl	12.4701	1.575
Mean corpuscular volume (MCV) fl	79.8475	12.678
Mean corpuscular hemoglobin (MCH) pg	26.0029	5.495
Mean corpuscular hemoglobin concentration (MCHC)gm/dl	31.5018	3.439

fl:femtoliters, pg:pictograms, gm/dl:grams per deciliter.

**Table-II:** Comparison of anthropometric and hematological variables between day scholars and hostilities using independent sample t test (n = 221).

*Variables*	*Hostilities (n=143) Mean (SD)*	*Day scholars (n=78) Mean (SD)*	*p-value
Age	19.89 (1.08)	19.97 (0.56)	0.006*
Height	62.29 (2.72)	62.24 (2.27)	0.233
Weight	55.19 (7.43)	59.16 (11.27)	0.000*
Body mass index (BMI)	22.15 (3.31)	23.66 (4.10)	0.853
Hemoglobin	12.29 (1.65)	12.79 (1.36)	0.029*
Mean corpuscular volume (MCV)	79.28 (13.03)	80.87 (12.02)	0.600
Mean corpuscular hemoglobin (MCH)	26.07 (5.99)	25.87 (4.47)	0.760
Mean corpuscular hemoglobin concentration (MCHC)	31.80 (1.74)	30.95 (5.26)	0.027*

* p-value≤ 0.05 is considered statistically significant values are given as Mean (SD).

Anemia was observed in 74 (33.4%) students out of total 221 MBBS students. 57(25.8%) students were having mild anemia (Hb% 10.0-11.9 gm/dL), 16(7.2%) had moderate anemia (7-9.9gmdl) and only 0.5% were severely anemic (≤ 7 gm/dl) Mean ± SD of MCV, MCH and MCHC of the anemic subjects were 78.36± 11.7, 24.82± 4.39 and 30± 1.161 respectively. Mean ± SD of MCV, MCH and MCHC of the normal subjects were 81± 13.10, 27± 5.89 and 32 ± 4.06. About 39.2% of hostelites were anemic as compared to 23.1% day scholar students which was proved to be significance at P value= 0.015* (Table-III).

**Table-III:** Severity of anemia among medical students.

*Anemia Grades*	*Hostilities (n = 143)*					*Day scholars (n=78)*				

	*Frequency (%)*	*Hb Mean (SD)*	*MCV Mean (SD)*	*MCH Mean (SD)*	*MCHC Mean (SD)*	*Frequency (%)*	*Hb Mean (SD)*	*MCV Mean (SD)*	*MCH Mean (SD)*	*MCHC Mean (SD)*
No anemia	87 (60.8%)	13.3 (0.77)	111.00 (13. 1)	26.7(6.6)	31.82(1.8)	60(76.9%)	13.3(0.83)	81.6(13.0)	26.3(4.6)	31.02(5.9)
Mild anemia	41 (28.7%)	11(0.57)	79(7.2)	25.2(3.32)	31(1.54)	16(20.5%)	10(0.64)	78(7.9)	24.2(3.4)	30.7 (1.75)
Moderate anemia	14 (9.8%)	9.1(0.47)	79(9.1)	26(3.4)	32(1.44)	2(2.6%)	9.4(0.35)	77(4.9)	23(2.3)	30.56(1.06)
Severe anemia	1 (0.7%)	7.4(0.26)	83(11.3)	26(5.5)	30(1.47)	0(0)	-	-	-	-
P values	0.000*	0.001*	0.81	0.53	0.700	0.002*	0.000*	0.60	0.19	0.97

Hemoglobin (Hb), Mean corpuscular volume (MCV), Mean corpuscular hemoglobin (MCH), Mean corpuscular hemoglobin concentration (MCHC) Hb cut offs were taken as per WHO criteria for detection of various grades of anemia^1^ANOVA was used to compare Hb and red cell indices levels among the grading of anemi X^2^for comparison of percentages. *p*-value≤ 0.05 is considered statistically significant values are given as Mean (SD).

On analyzing by BMI sub groups it was noticed that among 221 students of study population 11.31% (n=25) students were underweight, 33.03% (n=73) had normal BMI, and 55.6% (n=123) were overweight. On comparison of BMI between hostelites and day scholars it was noted that 14% of hostelites and 5.1% of dayscholars were under weight. About 32% of the hostelites had normal BMI as compared to day scholars in which33% had normal BMI. 52% of hostelites were overweight as compare to day scholars in which 61%were overweight.

On further analysis of anemic student’s weight it was observed that 56% of underweight, 23.2% of normal weight and 34.9% of overweight students were anemic. (P value=0.029*) (Table-IV). ANOVA indicates statistically significant difference in mean Hb among the BMI subgroups (P value =0.006*) (Table-V). Fig-I indicates the results of posthoc Tuckey’s test for multiple comparison which indicates the significant differences among underweight vs normal weight (P value=0.005*) and underweight vs overweight (P value = 0.017*) with respect to Hb. No significant differences in mean Hb was found among normal weight and overweight (P value = 0.629).

**Table-IV:** Status of anemia among students belonging to different nutritional levels.

*Variables*	*Underweight n = 25*	*Normal weight n = 73*	*Overweight n = 123*
Mild anemia	11 (44%)	10 (13.6%)	36(29.2%)
Moderate anemia	3 (12%)	7(9.5%)	6 (4.8%)
Severe anemia	0	0	1(0.5%)
Total anemic subjects	14(56%)	17(23.2%)	43 (34.9%)
p-value	0.029*		

* p-value ≤ 0.05 is considered statistically significant

**Table-V:** Comparison of serum hemoglobin levels between BMI groups using ANOVA.

*BMI groups*	*N*	*Mean*	*Std. Deviation*	*P value*
Underweight (< 18.5)	25	11.5720	1.34087	0.006*
Normal (18.5 – 23.0)	73	12.7164	1.67548
Overweight (> 23.0)	123	12.5065	1.50332

Total	221	12.4701	1.57501

* p-value ≤ 0.05 is considered statistically significant. Alpha 0.05, Confidence Interval 95%

**Fig-I:**
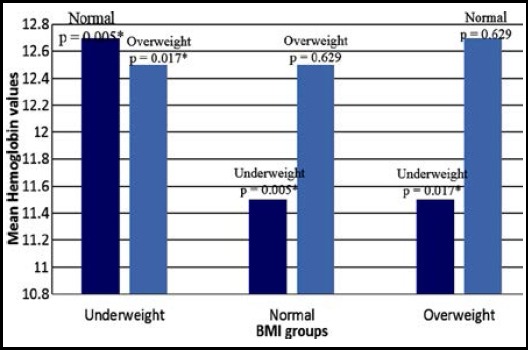
Multiple comparisons of serum hemoglobin levels of BMI groups using post hoc Tuckey’s test. P ≤ 0.05 is considered statistically significant.

## DISCUSSION

Anemia is the major health problem worldwide.^13^ High Prevalence of anemia among the young females is alarming due to its consequences on the health and productivity.^14^ Low Health status of young girls reflects gender discriminations right from their birth in our society. Inequitable distribution of health resources within household and society is the leading cause of nutritional anemia among the females.^14^ According to WHO most common cause of anemia is iron deficiency anemia. Iron plays a pivotal role in erythropoiesis.^13^ Prolonged negative iron balance due to insufficient dietary iron intake or poor bioavailability, increased requirements for iron during development and pregnancy and increased iron losses resulting from menstruation and worm infestations are contributing factors.^2^,^15^ It is estimated that 42mg of iron is lost per menstrual cycle as documented by various studies conducted in different areas which is the leading cause of anemia in females.^10^ Proper nutrition is required to provide this essential element and other micronutrients to reduce the risk of anemia. WHO is determined to reduce anemia globally by 50% till 2025.

Some countries including India, China, Venezuela and Vietnam have successfully implemented WHO strategies for prevention and control of anemia in reducing its prevalence but in Pakistan implementation on health strategies are required for itseradication.^2^ Many studies in the past were attempted to evaluate the prevalence of anemia in pregnancy, infants and adolescents but very few studies were conducted among the university students, who are the future of our country. For the planning and implementation of health strategies and policies for eradication of anemia, there is need to increase awareness among them about the effect of nutritional status on common health issues like anemia and its grave effects on learning, cognition, attention and behavior. This study was aimed to evaluate the nutritional status through BMI of female university students and to identify the students having anemia and to correlate it with BMI.^1^,^16^

Greater number of students of current study had mild anemia (25.8%), followed by moderate anemia (7.2%) and only 1(0.5%) student out of 221 was severely anemic. Morphologically it was microcytic hypochromic anemia as the red cell indices of anemic subjects were lower than normal levels. Most probably it was an iron deficiency anemia as the hemoglobinopathies were in exclusion criteria. Regarding the grading of anemia, Pandey S and his colleagues are in agreement to the report of current study^6^ They reported that 68.97% student were suffering from mild anemia, 31.03% had moderate anemia and no student were found to be severely anemic among their study population. Similar trend was documented by Chaudhary SM et al, who found 69.2% and 30.8% of subjects with mild and moderate anemia respectively and none of the subjects had severe anemia among their study population.^17^

Analysis by BMI categories in the present study showed that percentages of anemia was found to be high among underweight students (56%) as compared to normal (23.2%) and overweight students (34.9%). Studies attempted by various scientists have documented the similar findings.^1^,^6^,^10^ These findings suggest that good nutritional status reduces the risk of anemia. A large prevalence of anemia is attributed to nutritionally inadequate diet among the girls.^18^ On comparing the hostelites and day scholars, the greater numbers of anemic students were found in hostelites ((39.1%) as compared to day scholars(23.07%).

The mean weight of day scholars was 59.16 kg on the other hand this decreases to 55.19 kg in hostelites which show significance difference (P value = 0.000*). 14.6% of the hostelites were categorized as underweight as compared to day scholars it was 5.1%, most probably because of their poor eating habits which does not fulfill their nutritional requirements.^19^ Unhealthy eating practices among them including breakfast skipping, light meals, snacking and fast food consumption are recognizable facts.^10^,^20^ Lack of intake of fruits and vegetables in diet are also contributing factors for poor health status.^21^ Stress of hostel and burden of medical study would also negatively affect their diet.^22^ Previous studies conducted by researchers have reported that the medical students possess high risk for chronic illness due to poor eating practices.^23^

### Limitations

The sample size of this study is small and hence the results of this study could not be generalized to the whole population. Secondly the study was conducted in medical students of only one college. Additional tests such as serum ferritin, serum iron and transferrin or total iron binding capacity(TIBC) were required for diagnosis of iron deficiency anemia. Which was not estimated in this study showing the weakness of this study

### Suggestions

Young girls of age 18-25 years are more prone to get nutritional anemia. Routine checkup and hemoglobin estimation should be done frequently for the screening of anemia. Preventive programs and policies of the government can target this age group to reduce prevalence of anemia. WHO strategies for prevention and control of anemia should be implemented at government and organizational level to reduce its prevalence. Interventional programs at primary health-care systems and colleges should also be organized for improvement in the nutritional status of anemic students to reduce its complications. Iron supplementation is thus required for the target group.

## CONCLUSION

In summary, anemia is prevalent among medical students which might also affect the efficiency of these students. This study has highlighted the problem of anemia in medical students. Further studies should be conducted on a larger sample size to evaluate the cause and to eradicate this problem, further more hostelite students should take care of their diet and they should be encouraged to eat balanced meal in order to cater the problem of anemia.

### Authors` Contribution

***Dr. Shireen Jawed:*** Study design, data collection, Interpretation of results, manuscript writing

***Dr. Sundus Tariq:*** Statistical analysis, interpretation of results, formulation of tables, writing the manuscript. Reviewed and approved the manuscript.

***Dr. Saba Tariq:*** Acquisition of data, interpretation of results, editing and formatting the manuscript. Reviewed and approved the manuscript.

***Dr. Anwar Kamal:*** Study design, statistical analysis, interpretation of results, and formulation of tables. Reviewed and approved the manuscript.
